# A peptidoglycan recognition protein acts in whitefly (*Bemisia tabaci*) immunity and involves in *Begomovirus* acquisition

**DOI:** 10.1038/srep37806

**Published:** 2016-11-28

**Authors:** Zhi-Zhi Wang, Min Shi, Yi-Cun Huang, Xiao-Wei Wang, David Stanley, Xue-Xin Chen

**Affiliations:** 1 Ministry of Agriculture Key Lab of Agricultural Entomology, Institute of Insect Sciences, Zhejiang University, 866 Yuhangtang Road, Hangzhou 310058, China; 2 Biological Control of Insects Research Laboratory, Agricultural Research Service, U.S., Department of Agriculture, 1503 S. Providence Road, Columbia MO 65203, USA

## Abstract

Peptidoglycan recognition proteins (PGRPs) are multifunctional pattern recognition proteins. Here, we report that a PGRP gene, *BtPGRP*, encodes a PGRP from the whitefly *Bemisia tabaci* (MEAM1) that binds and kills bacteria *in vitro*. We analyzed *BtPGRP* transcriptional profiling, and the distribution of the cognate protein within the midgut. Fungal infection and wasp parasitization induced expression of *BtPGRP*. Silencing *BtPGRP* with artificial media amended with dsRNA led to reduced expression of a gene encoding an antimicrobial peptide, *B. tabaci c-type lysozyme*. B*egomovirus* infection also led to increased expression of *BtPGRP*. We propose that BtPGRP has a potential *Tomato yellow leaf curl virus* (TYLCV) binding site because we detected *in vitro* interaction between BtPGRP and TYLCV by immunocapture PCR, and recorded the co-localization of TYLCV and BtPGRP in midguts. This work addresses a visible gap in understanding whitefly immunity and provides insight into how the whitefly immunity acts in complex mechanisms of *Begomovirus* transmission among plants.

Insect innate immunity is assorted into three broad categories, physical, cellular and humoral, although they overlap in functions. The body surfaces, periotrophic matrices and alimentary canals are physical barriers to invading microbes; once the physical barriers are breached, epithelial cellular, hemocytic and humoral immune effectors are activated. Cellular immune reactions include phagocytosis, nodulation and, for larger invaders, encapsulation^1^,^2^. Cellular immunity is activated immediately an infection is detected and is responsible for clearing most infecting microbes from hemolymph circulation^3^. Humoral immune reactions include biosynthesis of antimicrobial peptides (mainly in fat body, but also in hemocytes and epithelia of other tissues), which appear in the hemolymph of infected insects about 6 to 12 h following infection. Haine *et al*.^4^ regarded cellular defenses as the ‘first line’ of defense and the AMPs as a ‘mop up’ function to clear remaining microbes that survived cellular defenses. Cellular and humoral defenses depend on surveillance by a microbial recognition system that includes pattern-recognition receptors capable of recognizing microbe-specific molecules, known as pathogen-associated molecular patterns^5^. These include lipopolysaccharide, peptidoglycans (PGN), β-1, 3-glucans, and lipoteichoic acids. PGN recognition proteins (PGRPs) comprise a family of proteins, first discovered from the hemolymph of the silkworm, *Bombyx mori,* as proteins that bind to bacterial PGN and activate the prophenoloxidase cascade^6^. PGRPs are present in invertebrates and vertebrates, but not in nematodes and plants. Almost 100 PGRP family members have been identified, all with at least one conserved PGRP domain homologous to bacteriophage. Insects have several PGRP genes. For example, *Drosophila melanogaster* has 13 PGRP genes encoding 19 proteins and the mosquito, *Anopheles gambiae,* has 7 PGRP genes encoding 9 proteins^7^,^8^. Based on the gene length and protein structure, PGRPs are assorted into two groups: short PGRPs (PGRP-S), small extracellular proteins (19–20 kDa) and long PGRPs (PGRP-L), which have long transcripts and can be extracellular, intracellular and membrane-spanning proteins^8^.

PGRPs are responsible for several actions in insect immunity. In *Drosophila*, and probably most insects, some PGRPs, such as PGRP-SA, act in sensing and distinguishing categories of infecting microbes (Gram-positive; Gram-negative; fungal) and activating down-stream immune effector pathways, such as the immune deficiency (imd) pathway and the Toll pathway^9^,^10^. Others, for example, the *Drosophila* PGRP-LF interacts with PGRP-LC to down-regulate the imd pathway^11^. Still other PGRPs have amidase activity. These proteins, including PGRP-SC and PGRP-LB, also act as negative modulators of the imd pathway, which protects flies from lethal excessive immune reaction to transient infection^12^. PGRPs also act in homeostasis of the *Drosophila* alimentary canal^2^,^13^, enabling the tolerance of indigenous microbes and elimination of pathogens. Taken with related findings in vertebrates, the PGRP family has a principal role in innate immune responses.

The whitefly *Bemisia tabaci* (Gennadius) is a species complex composed of at least 35 morphologically indistinguishable species^14^,^15^. Some members of the *B. tabaci* species-complex, especially the cryptic species Middle East-Asia Minor 1(MEAN 1) and Mediterranean, make up a serious pest complex in agricultural ecosystems. Aside from direct feeding damage, *B. tabaci* is an effective vector of plant pathogenic viruses^16^. Of the whitefly-transmitted virus species, approximately 90% belong to the genus *Begomovirus*, such as the *Tomato yellow leaf curl virus* (TYLCV), the *Tomato yellow leaf curl China virus* (TYLCCNV), and the *Tobacco curly shoot virus* (TbCSV)^17^. Begomoviruses are transmitted in a persistent circulative manner in which viruses move from gut into hemolymph and on to other tissues within their hosts^16^,^18^. Some *Begomovirus* species, TYLCV, for example, may replicate within the whitefly vector, while others, such as the tomato mottle virus, probably do not^19^,^20^. In general, Geminiviruses, including *Begomovirus*, replicate in their host plants^21^. Several factors operate in the transmission of *Begomovirus*. For example, a 63-kDa GroEL homolog produced by endosymbiotic bacteria has high binding affinity for TYLCV and may protect the virus from destruction during its passage through the hemolymph^16^. Two members of the heat-shock protein (HSP) family (HSP70 and BtHSP16) and a midgut protein (MGP) also bind begomoviruses *in vitro;* HSP70 may act in protecting the vector against begomoviruses while translocating within the whitefly^22^,^23^,^24^.

Several *B. tabaci* transcriptome analyses were designed to identify whitefly genes involved in *Begomovirus* transmission^25^,^26^,^27^,^28^. Despite considerable progress, however, the molecular and biochemical mechanisms underlying *Begomovirus* transmission, particularly mechanisms related to maintaining whitefly fitness, are not yet thoroughly elucidated. The situation is probably due to the diversity and complexity whitefly/virus relationships^16^.

We addressed a visible gap in understanding whitefly immunity by posing the hypothesis that a PGRP gene, *BtPGRP*, encodes a PGRP that binds and kills bacteria and that *Begomovirus* infection induces *BtPGRP* expression. Here, we report on the outcomes of experiments designed to test our hypothesis.

## Results

### Cloning and sequence analysis of *BtPGRP*

**Figure 1:**
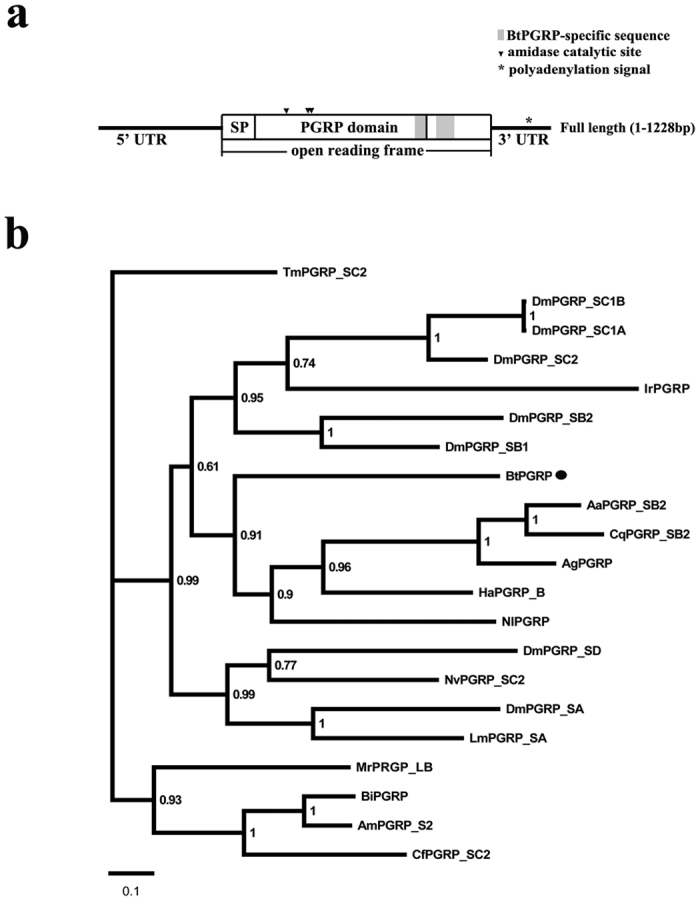
BtPGRP sequence information and phylogeny analysis. (**a**) Schematic representation of the full cDNA for BtPGRP. The solid line indicates the 5′- and 3′-untranslated regions. The white boxes show the coding region, with the signal peptide (SP) and PGRP domain. The grey box represents the BtPGRP-specific sequence. The triangle indicates the amidase catalytic site and polyadenylation signal (AATAAA) is marked with asterisk. (**b**) Phylogenetic analyses of BtPGRP with other insect PGRPs. Sequences were selected from NCBI databases. The numbers at each node are bootstrapping values and the bar indicates the scale of branch length. BtPGRP is marked with a solid circle. The accession number of the sequence in GenBank are as follows: Bt, *B. tabaci* (KJ868812); Dm, *D. melanogaster* (PGRP-SA: NP_572727; PGRP-SB1: CAD89138; PGRP-SB2: CAD89140; PGRP-SC1a: CAD89163; PGRP-SC2: CAD89187; PGRP-SD: CAD89197); Nv, *N. vitripennis* (XP_001603488); Tm, *T. molitor* (BAJ23047); Cq, *C. quinquefasciatus* (XP_001849091); Am, *A. mellifera* (NP_001157188); Ag, *A. gambiae* (XP_003435776); Aa, *A. aegypti* (XP_001654275); Nl, *N. lugens* (AEO89449); Ha, *Helicoverpa armigera* (AFP23116); Lm, *Locusta migratoria* (AFD54029); Mr, *Megachile rotundata* (XP_003703217); Bi, *Bombus ignitus* (ADD10756); Cf, *Camponotus floridanus* (EFN73970); Ir, *Ixodes ricinus* (JAA67198).

The full-length *BtPGRP* cDNA (GenBank accession number KJ868812) is 1228 bp, containing an open reading frame of 708 bp encoding a 235 amino acid protein (Fig. 1a). The predicted molecular weight (MW) of the mature protein is 22.67 kDa. The sequence was followed by a 3′untranslated stretch of 256 nucleotides containing a possible polyadenylation signal (AATAAA). By confirming the full length of *BtPGRP* sequence using both DNA and RNA of *B. tabaci* (MEAN1, MED and ZHJ1), we found that *BtPGRP* from the three cryptic species shared 99.9% identity. Multiple sequence alignment of the deduced amino acid sequence shows high similarity to other members of PGRP superfamily. The amino acids H^57^, T^182^, and S^184^ are the predicted amidase catalytic sites and H^57^ is a Zn^2+^ binding site motif. Phylogenetic analysis showed that BtPGRP from *B. tabaci* forms an orthologous group with other insect PGRPs (Fig. 1b).

### Expression and enrichment of recombinant BtPGRP (rBtPGRP)

We expressed an rBtPGRP in *E. coli* BL21 (DE3) cells. The products contained His•tag (6 aa) -Thrombin-T7•tag sites at the N-terminal. *E. coli* expression of the rBtPGRP was not induced in the absence of IPTG (Supplementary Fig. S1A, lane 1), and substantially induced in the presence of IPTG (Supplementary Fig. S1A, lane 2). Figure S1A, lane 3 shows the rBtPGRP was highly enriched on His TALON™ Gravity Columns, yielding about 2.5 mg of protein. The recombinant protein reacted with a rabbit His-tag antibody on western blot, yielding a band of approximately 28 kDa, the expected size of the highly enriched protein (Supplementary Fig. S1B).

### Microbial binding and bactericidal activity assays

**Figure 2:**
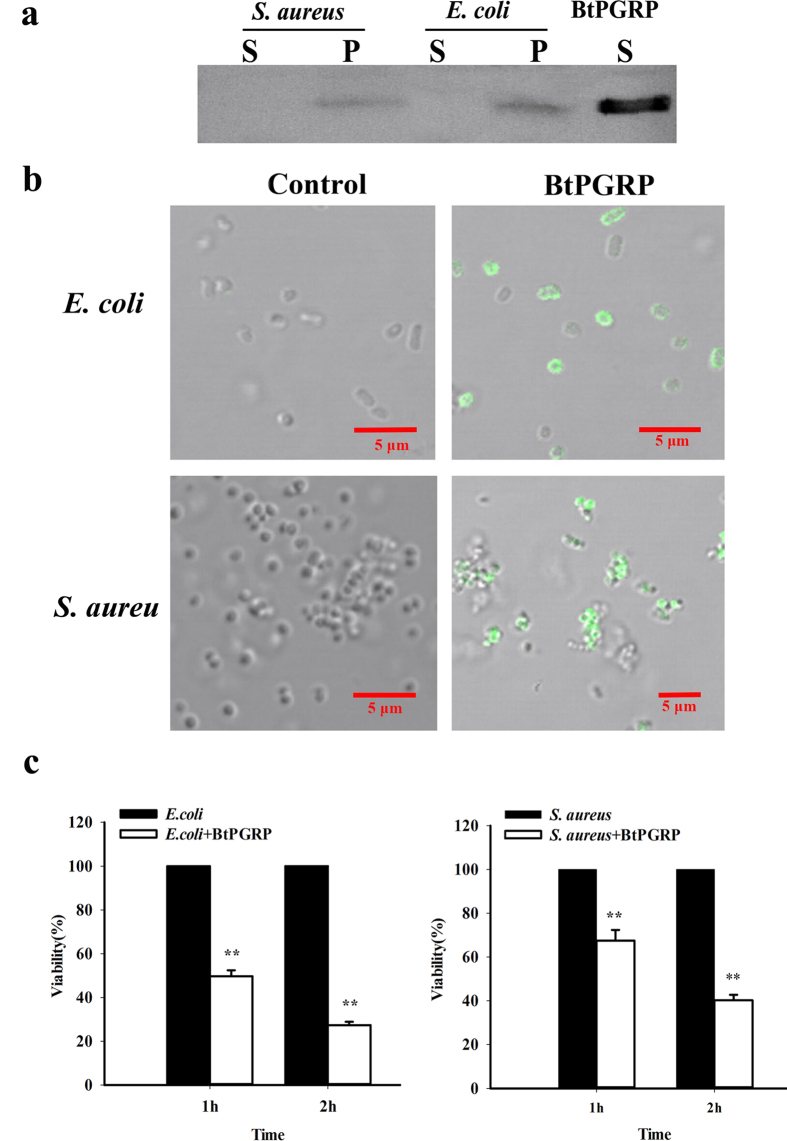
Microbial binding and bactericidal activity of rBtPGRP. (**a**) Western blot analysis of the microbial binding activity of rBtPGRP using an anti-BtPGRP antibody. Live *S. aureus* and *E. coli* were incubated with rBtPGRP for 10 min. Bound rBtPGRP (P) was separated from free rBtPGRP (S) in the supernatant by centrifugation. rBtPGRP without added microorganisms was used as a control (far right lane). (**b**) Immunofluorescence staining shows the binding of rBtPGRP to live bacteria. *E. coli* and *S. aureus* were treated with rBtPGRP for 10 min. rBtPGRP (green) is seen bound to the bacteria cell walls of *E. coli* and *S. aureus*. (**c**) Bactericidal activity of rBtPGRP against *E. coli* (left) and *S. aureus* (right). Diluted bacteria samples were incubated with rBtPGRP for 1 h or 2 h and then spread on LB agar plates. Viability was recorded as CFUs/ml after incubation for 18 h (n = 3). **P < 0.01.

Our western blot analysis shows rBtPGRP bound *E. coli* and *S. aureus* with high affinity (Fig. 2a, lanes P for both bacterial species) and fluorescence microscopy shows the rBtPGRP localized to the cell walls (seen as the green staining) of both bacterial species (Fig. 2b). We recoded aggregation of *S. aureus* after rBtPGRP treatment. The protein did not bind to the *C. albican* cell walls (Supplementary Fig. S2). Compared to the positive control enzyme, lysozyme, the refolded BtPGRP lacked amidase activity against insoluble PGN from *E. coli* and *S. aureus* (Supplementary Fig. S3), although it was strongly bactericidal to both tested bacteria (Fig. 2c). Compared with controls, 50% of *E. coli* was killed after 1 h incubation, which increased to 73% during the second hour. Similarly, 33% of *S. aureus* was killed after 1 h incubation and 60% after 2 h. In negative control experiments, the N-terminal tag peptide (His•tag (6 aa) -Thrombin-T7•tag) did not bind to bacteria and had no antibacterial activity (Fig. S4).

### *BtPGRP* gene expression

**Figure 3:**
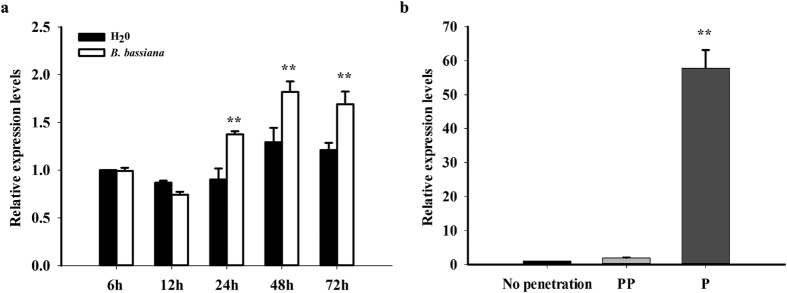
*BtPGRP* mRNA acumulation after *B. bassiana* infection (**a**) and wasp parasitization (**b**). No penetration: control nonparasitized instars; PP: pre-penetrated instars; P: penetrated by *E. hayati*. The data represent the mean ± SD of three independent biological replicates. **P < 0.01.

*BtPGRP* was constitutively expressed in all examined tissues, midgut, ovary, fat body and egg, and all developmental stages. There was relatively low expression in egg and 2^nd^ instar stages (Supplementary Fig. S5a). We found the expression profiles of *BtPGRP* were not influenced after *B. tabaci* fed on selected host plants (Supplementary Fig. S5b). After we applied *B. bassiana* to infect the white flies, *BtPGRP* expression was up-regulated after 24 h post-inoculation (Fig. 3a). With respect to the influence of parasitoids, *BtPGRP* expression increased 50-fold at the penetrated stage after *Eretmocerus hayati* parasitization (Fig. 3b).

### Expression of *BtPGRP* gene following dsRNA ingestion

**Figure 4:**
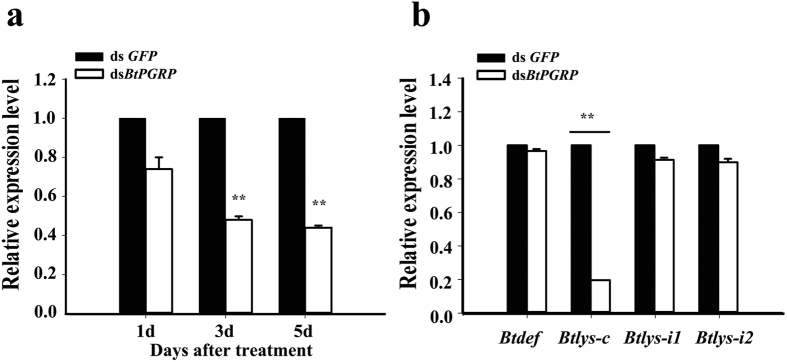
Dietary dsBtPGRP reduced accumulation of *BtPGRP* transcripts. (**a**) *BtPGRP* expression in adult whiteflies at indicated days following initiation of dsRNA feeding. (**b**) Accumulation of mRNAs encoding four AMPs 5 days after beginning *BtPGRP* dsRNA ingestion. The data represent the mean ± SD of three independent biological replicates. **P < 0.01.

The abundance of *BtPGRP* transcripts was substantially decreased (up to 50% at 3 days) after dietary exposure to dsRNA for 1, 3, and 5 days (Fig. 4a). At day 5, experimental whiteflies were analyzed for the expression of the selected AMP genes, *lysozyme (Btlys-C, Btlys-i1* and *Btlys-i2*) and *defensin (Btdef*). Knockdown of *BtPGRP* led to significantly decreased abundances of mRNA encoding *Btlys-C*, but not the other AMP genes (Fig. 4b).

### *Begomovirus* acquisition influenced *BtPGRP* expression

**Figure 5:**
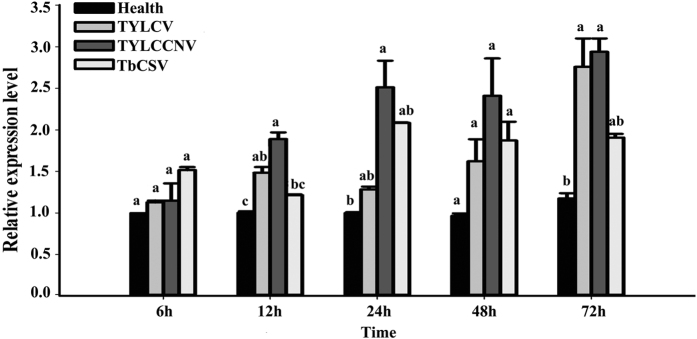
*Begomovirus* infection led to increased abundancers of *BtPGRP* transcripts in adult whiteflies. The data represent the mean ± SD of three independent biological replicates. Bars annotated with the same letter are not significantly different at *P* < 0.05.

Newly emerged whiteflies were released onto the leaves of healthy tomato plants in one cage and, separately, onto virus-infected (TYLCV-, TYLCCNV-, or TbCSV-infected) tomato plants in other cages. Two cages of nonviruliferous and viruliferous whiteflies were randomly collected at the end of 5 acquisition periods (6 h, 12 h, 24 h, 48 h and 72 h). Figure 5 shows the BtPGRP expression was significantly higher from 12 h to 72 h after acquisition of TYLCV and, separately, TYLCCNV. Compared with control, nonviruliferous whiteflies, the expression level was increased by 3-fold at 72 h after acquisition. The highest *BtPGRP* expression after acquisition of TbCSV, up by 2-fold occurred at 48 h. Although it appears otherwise, these multi-fold increases were significant, except for the influence of TbCSV.

### BtPGRP interacts with TYLCV

**Figure 6:**
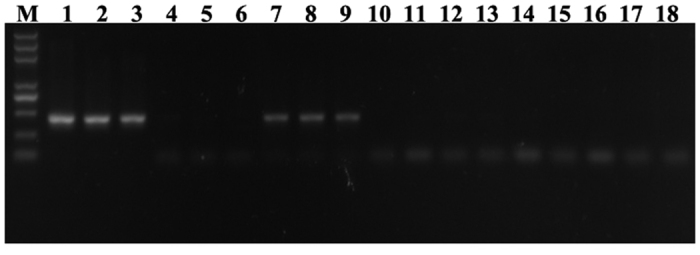
BtPGRP-TYLCV CP interaction in viruliferous whiteflies. TYLCV-specific PCR products were obtained after viruliferous whiteflies were applied to PCR tubes coated with BtPGRP antibody (lanes 7–9), as seen in tubes coated with TYLCV CP antibody (lanes 1–3). Negative controls, applying viruliferous whiteflies to uncoated tubes are shown in lanes 4–6, and applying extracts from nonviruliferous whiteflies to coated tubes are shown in lanes 10–18.

We used a IC-PCR assay to validate the interaction between TYLCV and BtPGRP. TYLCV-specific PCR products were obtained after viruliferous whiteflies were applied to PCR tubes coated with an anti-BtPGRP antibody (Fig. 6, lane 7–9) as also seen with tubes coated with an anti-TYLCV CP antibody (Fig. 6, lane 1–3). Negative control experiments with tubes not coated with the antibody yielded no TYLCV PCR products after analysis of viruliferous whiteflies (Fig. 6, lane 4–6) or in analysis of extracts from nonviruliferous whiteflies (Fig. 6, lane 10–18).

### Co-localization of BtPGRP and TYLCV in *B. tabaci* midgut

**Figure 7:**
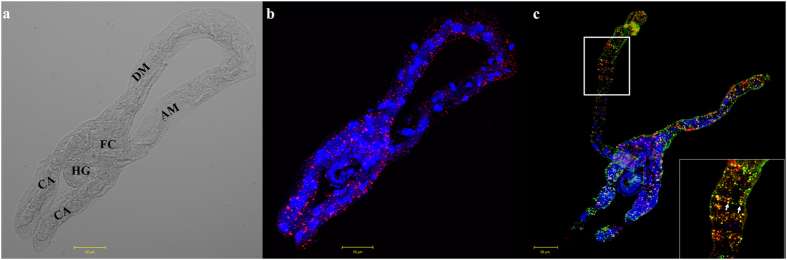
Co-localization of BtPGRP and TYLCV in midguts of viruliferous whiteflies. (**a**) The *B. tabaci* midgut anatomy. AM, ascending midgut; CA cecae; DM, descending midgut; FC, filter chamber; HG, hindgut. (**b**) Fluorescent immunolocalization of BtPGRP in midgut of *B. tabaci.* The red spots represent BtPGRP immunolocalization and the blue staining shows nuclei stained with DAPI. (**c**) Co-localization of BtPGRP and TYLCV in midguts of viruliferous whiteflies. The red spots indicate BtPGRP; green indicate TYLCV and yellow patches indicate co-localization of TYLCV and BtPGRP. Blue indicates DAPI staining of the nuclei.

We investigated the distribution of BtPGRP in *B. tabaci* midguts using a polyclonal anti-BtPGRP antibody. Immunofluorescent microscopy confirmed that BtPGRP is present throughout the midgut (Fig. 7a,b), extracellular to the midgut cells (Fig. 7b). We performed localization studies using an immunological detection of BtPGRP and TYLCV viral DNA in midguts dissected from viruliferous whiteflies. The presence of BtPGRP is indicated by red fluorescence and can be seen in each part of the midgut (Fig. 7c). The presence of TYLCV is shown by green fluorescence, observed in the whole midgut. Co-localization of BtPGRP and TYLCV is seen as yellow patches in the ascending midgut.

## Discussion

The data reported in this paper strongly support our hypothesis that a PGRP gene, *BtPGRP*, encodes a PGRP that binds and kills bacteria and that *Begomovirus* infection, except for TbCSV, induces *BtPGRP* expression. Our results form a sound argument. First, the nucleotide and calculated amino acid sequences of *BtPGRP* are similar to known *PGRP*s. Second, our phylogenetic analysis shows the *BtPGRP* fits into the insect *PGRP*s, with closest connection to another hemipteran, the brown planthopper. Third, we cloned the BtPGRP and expressed it in competent *E. coli* cells. The rBtPGRP protein bound to *E. coli* (Gram negative) and *S. aureus* (Gram positive) cells, with potent bactericidal activity. Fourth, *BtPGRP* is expressed in several tissues and all life stages from egg to adult, and is up-regulated after fungal infection and wasp parasitization. Fifth, a dietary dsRNA construct effectively reduced *BtPGRP* expression from days 3 to 5. This indicates *BtPGRP* acts in whitefly immunity. Sixth, exposure to separate plant pathogenic viruses (TYLCV, TYLCCNV and TbCSV) showed that TYLCCNV infection led to a time-dependent increase in *BtPGRP* expression up to 72 h post exposure. Finally, immunohistochemical protocols revealed the co-localization of BtPGRP protein and TYLCV within the midguts of viruliferous whiteflies. Taken together, each element of our results amounts to a convincing demonstration at least one *BtPGRP* operates in whitefly immunity and in *Begomovirus* vector capacity.

*BtPGRP* encodes a signal peptide, with no transmembrane domain, and is likely a secreted protein, which would place it in the short PGRP family (a PGRP-S). Multiple sequence alignment of the deduced amino acid shows that BtPGRP is similar to PGRPs in other insect species and contains a conserved PGRP domain at the C-terminus. Aside from assorting PGRPs into short- and long-transcript categories, some, but not all, of these proteins express amidase activity. With respect to phylogenetics, BtPGRP is an ortholog of PGRP -SB/ -SC in *D. melanogaster*. Although *Drosophila* PGRP-SB1/-SB2 and PGRP-SC1/-SC2 are thought to have conserved the amidase function^29^,^30^, BtPGRP lacks amidase activity, owing to the substitution of critical amino acids in the amidase-related motifs. The refolded BtPGRP did not have amidase activity against insoluble *E. coli* and *S. aureus*, confirming our inference. This is similar to *Drosophila* non-catalytic PGRPs (PGRP-SA, -SD, -LE, -LD or –LC), which function as pattern recognition receptors for PGN^31^,^32^,^33^,^34^. We identified only one S-type PGRP in whitefly, which stands in contrast to *D. melanogaster*, in which many S-type PGRPs, with varying roles, have been identified^35^,^36^. We infer from the orthology between BtPGRP and *Drosophila* PGRP -SB/-SC that BtPGRP functions in multiple roles in whitefly immunity as a combination of multiple S-type PGRPs, a concept we will test in future research.

Unlike *Drosophila* PGRP-SB/-SC, the BtPGRP contains an Arg^106^, which is associated with recognizing DAP-type PGN, as seen in the *Drosophila* PGRP-LE and PGRP-LC^37^. The Arg^106^ does not restrict BtPGRP binding exclusively to DAP-type PGN because it binds both Gram-negative and -positive bacteria. Previous studies have also implied that in addition to PGN, other envelope components such as beta-glucan, LPS and LTA, may be recognized by PGRPs^38^,^39^. BtPGRP reduced populations of *E. coli* and *S. aureus*, from which we infer that BtPGRP has antibacterial activity against both Gram-negative and Gram-positive bacteria.

The antibacterial activity is not directly correlated with amidase activity. In our study, BtPGRP showed no amidase activity against *E. coli* or *S. aureus* but had strong antibacterial activity to both bacterial classes. This is similar to some mammalian PGRPs, such as the human PGLYRP3 and PGLYRP4^40^. These PGRPs are bactericidal, but they are most likely not amidase-type PGRPs, as they do not have all five conserved catalytic residues. Other PGRPs also show bactericidal activity, such as *Drosophlia* PGRP-SB1^30^, *Bombus ignitus* PGRP-S^41^, *Helicoverpa armigera* HaPGRP-B and HaPGRP-C^38^ and the amphioxus *Branchiostoma japonicum* PGRP-S^39^. It is not clear how these PGRPs function but we speculate they constitute a new class of bactericidal proteins with structures and action mechanisms that differ from the currently known AMPs.

Many insect PGRPs are expressed in immune conferring organs and tissues, fat body, gut and hemolymph. For example, the *Drosophlia*, PGRP-SA, PGRP-SB and PGRP-SD are primarily present in hemolymph and expression is induced in fat body and PGRP-SC is induced in gut^9^. BtPGRP is constitutively expressed in all life stages and in the three tissues we analyzed, fat body, midgut and ovaries. AMPs, a component of insect humoral immunity, are produced in fat body. Insects also express epithelial immunity, recorded in salivary glands, Malpighian tubules and tracheal epithelia^3^. Ovaries represent the next generation and *Drosophil*a ovaries up-regulate expression of the AMP, attacin, when ovarian tumors are detected^42^. The idea that *BtPGRP* acts in immune functions is bolstered by results of infection and parasitization and by the outcomes of dietary dsRNA experiments. Fungal infection and wasp parasitization led to upregulation of *BtPGRP* mRNA expression, from which we infer the BtPGRP protein acts in one or more roles in white fly immunity. Dietary dsRNA led to reduced *BtPGRP* mRNA accumulatoin, which translated into a severe reduction in mRNA encoding an AMP, *Btlys-c*. BtPGRP may specifically influence *Btlys-c,* because it did not influence other AMP-encoding genes, *Btdef, Btlys-il* or *Btlys-i2* in immunologically naïve whiteflies. The overall picture indicates that BtPGRP acts in multiple immune-related functions.

Our data document co-localization of BtPGRP with TYLCV in the midgut. The IC-PCR aasay indicates a specific interaction between BtPGRP and TYLCV during TYLCV acqusition. We infer BtPGRP has a TYLCV binding site to form a BtPGRP and TYLCV complex. This is consistent with other studies reporting viral-associated up-regulation of PGRPs. *Drosophila* C virus infection led to increased PGRP-SA expression and SIGMAV infection resulted in increased expression of PGRP-SB1 and PGRP-SD in *Drosophila*^43^. Similarly, Gao *et al*.^2^ reported that *B. mori* cytoplasmic polyhedrosis virus infection led to inceased expression of another PGRP gene, *BmPGRP-S3*. Overall, we infer that BtPGRP acts in multiple immune-response functions.

## Materials and Methods

### Insects, host plants and microorganism strains

Our whitefly rearing procedure followed^27^. Adults of *B. tabaci* MEAM1 were released onto cotton plants and maintained for five generations in the lab at 26 ± 1 °C, 60% ± 10% relative humidity and natural photoperiod. Cotton plants were individually grown in plastic pots in greenhouses and no chemicals were applied during the growth and test periods.

Clones of TYLCCNV isolate Y10 (GenBank accession number AJ319675) and its DNAβ (GenBank accession number AJ421621), TYLCV isolate SH2 (GenBank accession number AM282874) and TbCSV isolate Y35 (GenBank accession number AJ420318) were agroinoculated into 3–4 true-leaf stage of tomato (*Solanum lycopersicom* L. cv. Hezuo903) as described by^44^. Viral infection of test plants was verified by the typical viral symptoms and confirmed by PCR using the procedure described by^45^. All plants were grown in a greenhouse under natural lighting at 25 °C.

*Escherichia coli* strain BL21 (DE3) was used as the expression host. *E. coli* DH5α was used as the host for sub-cloning and plasmid amplification. *E. coli* DH5α, *Staphylococcus aureus* and *Canidia albican*s were used to assay the activity of the purified rBtPGRP as representative of Gram-negative and -positive bacteria and fungi.

### RNA isolation and rapid amplification of cDNA ends (RACE)

An EST sequence homologous to *PGRP* was obtained from the *B. tabaci* transcriptome databases (MEAN1, MED and ZHJ1) by local blast using known insect PGRP sequences as queries. To get the full length of the *PGRP* cDNA sequence, total RNA was isolated from about 200 *B. tabaci* adults using SV Total RNA Isolation System (Promega). RNA yield was determined on a NanoDrop 2000 spectrophotometer (Thermo Scientific). 3′ and 5′ RACE were performed with the SMART RACE cDNA Amplification kit (Clontech) according to the manufacturer’s instructions. Specific primers (Supplementary Table S1) were designed on the basis of the EST sequence. The resultant PCR products were sub-cloned into pMD-19 vector (Takara) and then sequenced by Sangon Biotech (Shanghai, China).

### Expression, enrichment and refolding of BtPGRP recombinant proteins

The cDNA fragment encoding BtPGRP, without a signal peptide, was amplified by PCR primers with *BamH* I and *Hind* III restriction sites (Takara) (Supplementary Table S1). Products of the PCR assembly were ligated into the pET-28a vector (Novagen) using T4 DNA ligase (Promega). The resulting vector was transformed into the *E. coli* BL21 (DE3) for DNA sequence analysis. In our standard protocol, a single colony of *E. coli* BL21 (DE3) cells was grown to OD600 = 0.6 and induced by isopropyl -β – D-1- thiogalactopyranoside (IPTG) at a final concentration of 1 mM (Sigma). After 3 h induction, the bacterial cells were harvested by centrifugation and protein expression analyzed on 12% SDS-PAGE. We confirmed identity of the rBtPGRP by western blotting using a His-Tag HRP-conjugated rabbit polyclonal antibody (HuaAn Biotechnology, Hangzhou, China) as the primary antibody. His TALON™ Gravity Columns (Clontech) were used to highly enrich the rBtPGRP.

The proteins were refolded using a Float-A-Lyzer G2 (Spectrumlabs) dialysis device. The rBtPGRP solution was dialyzed (with agitation) for 4 h, followed by dialysis in a succession of buffers (Supplementary Table S2). The refolded protein was subjected to a final dialysis against 25 mM Tris (pH 7.0) containing 10 mM NaCl and 5% glycerol overnight and then ultra-filtered with Amicon Ultra-4 (*Millipore*). The refolded rBtPGRP was analyzed on a 12% SDS–PAGE gel followed by western blotting with the His-tag antibody (HuaAn Biotechnology). The concentration of rBtPGRP was measured by absorbance at 280 nm and then stored at −80 °C until use.

A polyclonal antibody was prepared by injecting rBtPGRP into rabbits by BGI. A rabbit Immunoglobulin G ELISA Kit (BGI, China) was used to evaluate the reactivity and specificity of the polyclonal antibodies, which were stored at −80 °C until use.

### *In vitro* assays of rBtPGRP activity

To check the binding specificity*, E. coli* and *S. aureus* were grown in MH broth. *C. albicans* was grown in a modified Martin Broth. When grown to logarithmic phase (OD600 = 0.6), 4 ml of *E. coli, S. aureus* and *C. albicans* were harvested by centrifugation at 3500 × g for 10 min, washed three times with PBS and resuspended in 40 μl of rBtPGRP (2 μg) in PBS. After 10 min incubation at room temperature, the suspensions were centrifuged and the pellets were washed and resuspended in 40 μl of PBS. Samples of pellets and supernatants were subjected to 12% SDS-PAGE and western blot analysis using the antiserum against BtPGRP (diluted 1:500 (v/v) in PBS) as just described.

Amidase activity was measured as described by^46^ with slight modification. rBtPGRP was incubated with insoluble PGN from *E. coli* and *S. aureus* (InvivoGen), and OD540 nm was recorded every five minutes over a 120 min period. Egg white lysozyme [EC 3.2.1.17] (Sangon, China) was used as positive control. Three independent biological replicates were performed.

For the bactericidal assay, bacterial cultures were grown in MH medium at 37 °C to an OD600 = 0.6. Four ml of *E. coli* and *S. aureus* were pelleted by centrifugation at 3500 × g for 5 min, washed twice with PBS and resuspended in PBS. Bacteria were diluted to 10^4^ and incubated with rBtPGRP (40 μg/ml) for 1 h or 2 h. The samples were spread on LB agar plates. The plates were incubated at 37 °C for 18 h and the number of colonies was counted. CFUs per ml are shown as the mean of three independent biological replicates. For the microbial binding and bactericidal activity assays, the N-terminal tag peptide (His•tag (6 aa) -Thrombin-T7•tag) was synthesized and used as a control.

### Quantitative real time PCR (qPCR)

qPCR assays were conducted following^47^. The reactions were conducted on a BioRad CFX96 qPCR System (BioRad, USA) using SYBR Green Real Time PCR Master Mix-Plus (TOYOBO, Japan) following the manufacturer’s instructions. The cycling program was 5 min at 95 °C; 40 cycles of 15 s at 95 °C and 35 s at 56 °C. β-*actin* was used as the reference gene^19^. Plasmid DNA encoding BtPGRP or β-*actin* were used to generate standard curves. Three independent reactions were performed for each sample. Data shown are the mean of three independent biological replicates. Relative expression values were calculated after normalizing to the maximum expression value. BtPGRP mRNA abundances were analyzed by one-way ANOVA, at *P < *0.05.

### *BtPGRP* mRNA abundances

Tissue samples, midgut, ovary and fat body and whole insects, including eggs, 1^st^–4^th^ instar larvae and adults, were separately analyzed by qPCR using primers listed in Table S1.

The influence of *B. bassiana* infection and *E. hayati* parasitization on the relative accumulation of *BtPGRP* transcripts was determined. For fungal infection, we followed the protocol of^48^. For whitefly parasitization, whiteflies in the second to third stage of nymph development were used for parasitization and two stages of parasitization were compared with developmental stage-matched non-parasitized nymphs. The samples for the experiments were taken according to^49^.

### dsRNA synthesis and RNAi

*BtPGRP* transcriptional templates were produced from total whitefly cDNA using gene-specific primers. The extended sequence of T7 polymerase promoter (5′-TAATACGACTCACTATAGG -3′) was fused with gene-specific primers at the 5′-end, and two separate PCR reactions with single T7 promoter were required to generate two separate single promoter temples. PCR products were purified using a PCR DNA Clean-up Kit (Axygen, USA) and used as template for *in vitro* transcription. dsRNA was synthesized using the T7 RiboMAX Express RNAi System (Promega, USA). Sense and antisense strands were transcribed from the DNA template in the same reaction. dsRNA was suspended in nuclease-free water, analyzed on 1% agarose gels and quantified by NanoDrop 2000 spectrophotometry. The dsRNA was stored at −80 °C until use. A 700 bp *GFP* (GenBank accession number U76561) (Clontech, USA) was used to synthesize *dsGFP* for a negative control.

Newly emerged whiteflies were collected into plastic tubes (5 × 7 cm). The tube openings were covered with two layers of parafilm, and 200 μl solutions containing 30 μg dsRNA were placed into the gap between the two layers. Parafilm was pre-treated with 0.1% diethylpyrocarbonate (DEPC) solution to remove any RNases, and then RNase free water was used to clean the DEPC from the parafilm. The dsRNA solution was changed every 24 h. *BtPGRP* mRNA expression after dietary dsRNA was analyzed by qPCR by using specific primers (Supplementary Table S1). The influence of dietary dsRNA on expression of genes encoding AMPs, defensin and three lysozymes were analyzed, also using gene-specific primers.

### Immunocapture-PCR (IC-PCR) assay

Interaction between BtPGRP and TYLCV was detected by IC-PCR using anti-BtPGRP polyclonal antiserum following^50^. The buffers used for IC-PCR are described by^51^. PCR tubes were filled with 200 μl of antiserum (1:500 dilution), incubated 3 h at 37 °C, and washed 5 times for 5 min/wash with 200 μl washing buffer. Homogenates from 10–20 whiteflies and from whiteflies caged with TYLCV-infected tomato plants for 48 h were incubated overnight at 4 °C in the coated PCR tubes in 200 μl of extraction buffer. The tubes were washed 5 times, 5 min/wash with 200 μl washing buffer and dried. PCR amplification of the viral DNA from the TYLCV virions bound to the PGRP protein was performed with the TYLCV-specific primers V61 and C473^52^, with three independent biological replicates.

### Immunofluorescence analysis of BtPGRP and TYLCV in midgut

Midgut tracts were isolated from adults (n = 30) in dissection buffer under a stereomicroscope, washed twice with PBS and fixed in 4% paraformaldehyde at 4 °C overnight. Tissues were washed gently three times, 5 min/wash, with PBST (1 X PBS with 0.1% Tween-20), permeabilized and blocked with 0.5% Triton X-100 in PBST containing 5% fetal bovine serum for 2 h at 37 °C. The midguts were incubated with rabbit anti-BtPGRP polyclonal antiserum (1:50 dilution) for 3 h at 37 °C, and then washed three times with PBST. The final incubation, 3 h with secondary antibody, Alex-flour 546 nm donkey anti-rabbit IgG (Invitrogen) (1:400 dilution), made the BtPGRP visible. The tissues were washed three times with PBST. Midguts were transferred onto regular microscopic slides, whole mounted in 80% glycerol supplemented with 1 μl of 10 mg/ml 4′,6-diamidino-2-phenylindole (DAPI) solution, and analyzed using a LSM 780 confocal microscope (Zeiss). Negative control midguts were similarly handled, after incubation without the BtPGRP antibody.

For co-localization analysis, TYLCV and BtPGRP, whitefly adults were allowed 48 h for TYLCV acquisition, then were isolated and treated as just described. Two primary antibodies (BtPGRP antibody, 1:50 dilution; TYLCV CP antibody, 1:100 dilution) and two secondary antibodies were applied. To visualize TYLCV, we used Alexa Fluor 488 nm donkey anti-mouse IgG, dilution 1:50 and to visualize BtPGRP we used Alexa Fluor 546 nm donkey anti-rabbit IgG, dilution 1:100. The specimens were examined on a LSM 780 confocal laser scanning microscope.

## Additional Information

**Accession codes:** Transcript sequences from this study can be accessed through NCBI TSA database accession number GCZW00000000. cDNA sequences: GenBank accessions KJ868812.

**How to cite this article**: Wang, Z.-Z. *et al*. A peptidoglycan recognition protein acts in whitefly (*Bemisia tabaci*) immunity and involves in *Begomovirus* acquisition. *Sci. Rep.* **6**, 37806; doi: 10.1038/srep37806 (2016).

**Publisher's note:** Springer Nature remains neutral with regard to jurisdictional claims in published maps and institutional affiliations.

## Supplementary Material

Supplementary Tables and Figures
