# Supplementary material for: A peptidoglycan recognition protein acts in whitefly (Bemisia tabaci) immunity and involves in Begomovirus acquisition
Source: Sci Rep. 2016 Nov 28;6:37806. doi: 10.1038/srep37806 (PMC5124967; doi:10.1038/srep37806)
Supplement: Supplementary Tables and Figures [file srep37806-s1.pdf]

Supplementary Information

A peptidoglycan recognition protein acts in whitefly (*Bemisia tabaci)* immunity and involves in Begomovirus acquisition

Zhi-Zhi Wang [a], Min Shi [a], Yi-Cun Huang [a], Xiao-Wei Wang [a], David Stanley[b] and Xue-Xin Chen[a]*

[a] *Ministry of Agriculture Key Lab of Agricultural Entomology, Institute of Insect Sciences, Zhejiang University, 866 Yuhangtang Road, Hangzhou 310058, China*

[b] *Biological Control of Insects Research Laboratory, Agricultural Research Service, U.S. Department of Agriculture, 1503 S. Providence Road, Columbia MO 65203, USA*

***Corresponding author:** *xxchen@zju.edu.cn, phone number: +86 571 88982868*

Table S1. Primer sequences used in this paper

**Primer name**	**Primer sequences（5'-3'）**	**Use**
PGRP-F 1	CACCACTCCCGACTTCCACCTTGC	3'RACE
PGRP-F 2	CTCGTGGGCTACTCGGAGCAGGAC	3'RACE
PGRP-R 1	AAGGTGGAAGTCGGGAGTGGTGGAT	5'RACE
PGRP-R 2	CGCCGAGAAATGCTATGTTGATGC	5'RACE
PGRP-BamH	CGGGATCCATTGAGGGTCGCCGAGATTCGTGGTACG	Vector construction
PGRP-Hind III	CCCAAGCTTCTATTCGACGAGGAGGGCGAG	Vector construction
V61	ATACTTGGACACCTAAT GG	IC-PCR
C473	AGTCACGGGCCCTTACAA	IC-PCR
actin-F	TGGAGATGGTGTTTCCCACAC	qRT-PCR
actin-R	CCAGCCAAGTCCAAACGAAG	qRT-PCR
q*PGRP*-R	TTTCGTGGATTTCTTTGC	qRT-PCR
q*PGRP*-F	CAAGGTGGAAGTCGGGAG	qRT-PCR
ds*PGRP*-F	GAGGCGATGGATCTGTTTAT	RNAi
ds*PGRP*-R	GGTGGCTGAACAGGGAGGAC	RNAi
PGRP-T7-F	ggatccTAATACGACTCACTATAGGGAGGCGATGGATCTGTT	RNAi
PGRP-T7-R	ggatccTAATACGACTCACTATAGGGGTGGCTGAACAGGGAGG	RNAi
ds*GFP*-F	AAGGGCGAGGAGCTGTTCACCG	RNAi
ds*GFP*-R	CAGCAGGACCATGTGATCGCGC	RNAi
GFP-T7-F	ggatccTAATACGACTCACTATAGGAAGGGCGAGGAGCTGTTC	RNAi
GFP-T7-R	ggatccTAATACGACTCACTATAGGCAGCAGGACCATGTGATC	RNAi

Table S2. Dialysis buffers used in succession to refold rBtPGRP.

Buffer No.	Buffer recipe
**1**	25mM Tris buffer (pH 8.0) containing 0.2M NaCl, 5mM DTT, 1mM EDTA and 6M urea
**2**	25mM Tris buffer containing 50mM NaCl, 1mM EDTA, 4M urea, 0.1mM l-arginine (Arg), 0.2mM oxidized glutathione (GSSG) and 2mM reduced glutathione (GSH) at pH 8.0
**3**	25mM Tris buffer containing 20mM NaCl, 1mM EDTA, 2M urea, 0.1mM Arg, 0.2mM GSSG and 2mM GSH at pH8.0
**4**	25mM Tris buffer containing 10mM NaCl, 1mM EDTA, 1M urea, 0.1mM Arg, 1mM DTT, 0.2mM GSSG and 2mM GSH at pH 7.4
**5**	25mM Tris buffer containing 10mM NaCl, 1mM EDTA, 0.5M urea, 0.1mM Arg, 1mM DTT, 1% Gly, 0.2mM GSSG and 2mM GSH at pH 7.4

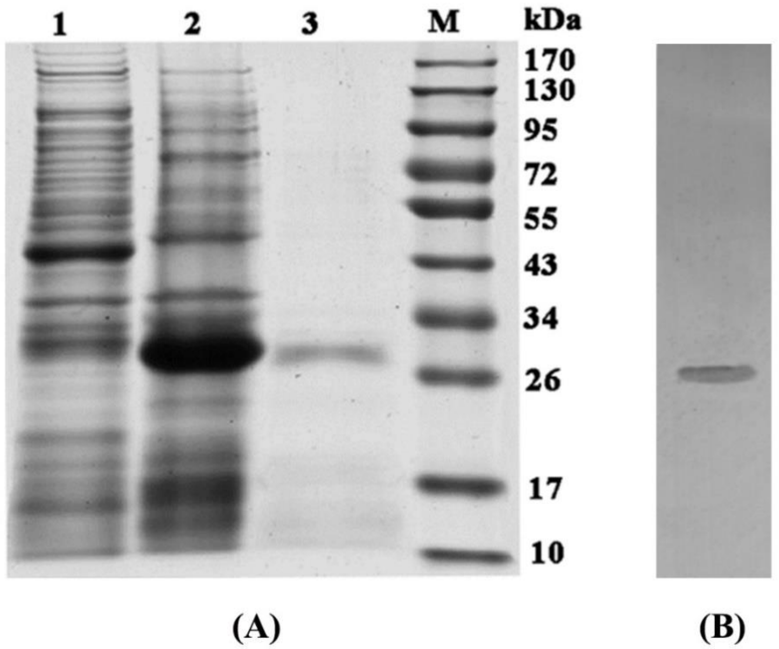

Figure S1. Expression of the BtPGRP gene in E. coli and purification of the BtPGRP peptide. (A) Expression of the BtPGRP gene in *E. coli* B121 (DE3) and enrichment of the rBtPGRP protein, analyzed on 12% SDS-PAGE gels. Lane M: protein molecular weight markers; Lane 1: the total proteins in uninduced *E. coli* Bl21; Lane 2: the expressed product of pET 28a-BtPGRP induced with 1 mM IPTG; Lane 3: Highly enriched recombinant protein. **(B)** Western blot analysis of purified rBtPGRP.

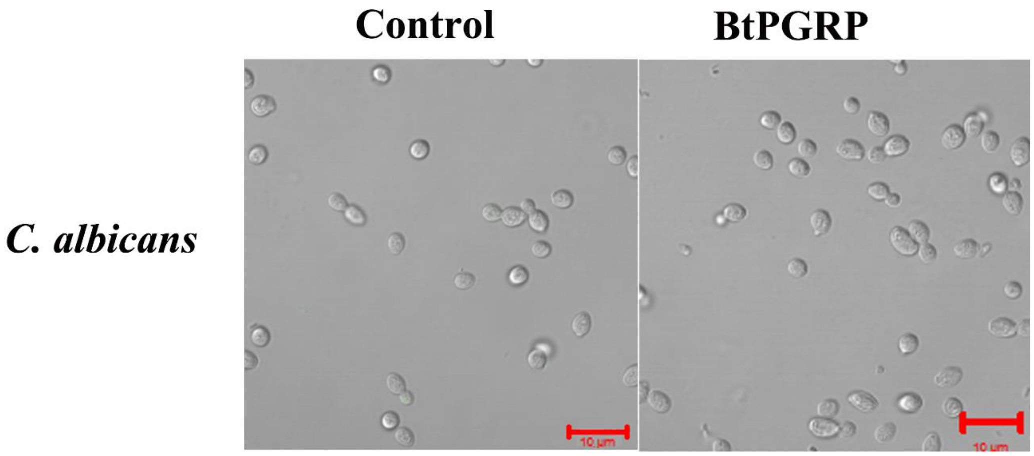

Figure S2. Immunofluorescence staining shows the binding of rBtPGRP to *C. albicans*. *C. albicans* was treated with rBtPGRP for 10 min. rBtPGRP (green) is not seen bound to the fungal cell walls of *C. albicans*.

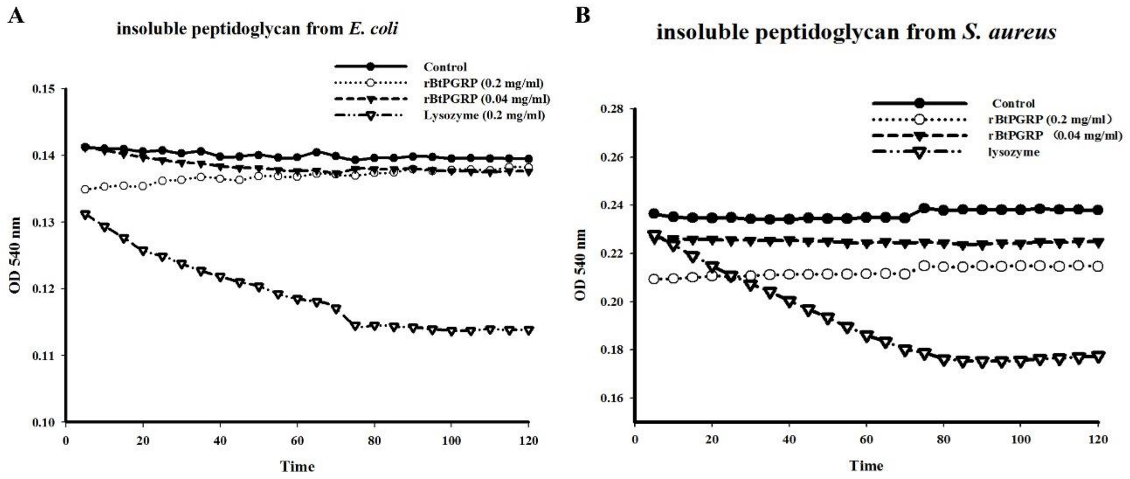

Figure S3. Enzymatic degradation of peptidoglycan. rBtPGRP or egg white lysozyme was incubated with insoluble peptidoglycan from *E. coli* and *S. aureus* (InvivoGen, USA), and the optical density (OD) at 540nm was recorded every fifth minute during a 120 min period.

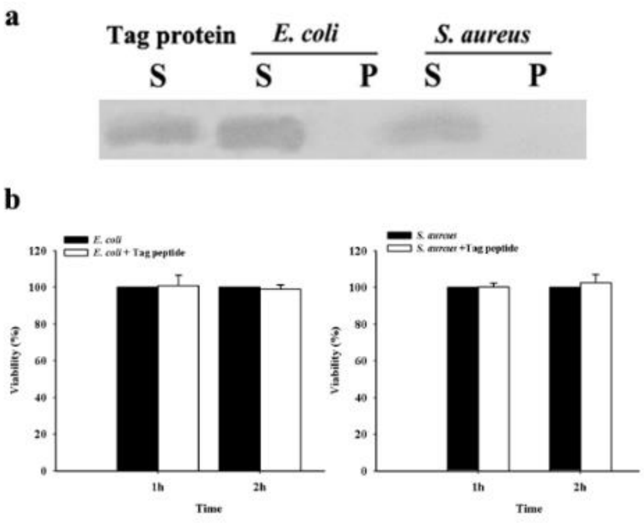

Figure S4. Microbial binding and bactericidal activity of His -Thrombin tag peptide. (a) Western blot analysis of the microbial binding activity of tag protein. Live *S. aureus* and *E. coli* were incubated with tag protein for 10 min. Bound tag protein (P) was separated from free tag protein (S) in the supernatant by centrifugation. Tag protein without added microorganisms was used as a control (left lane). The samples were analyzed by western blotting using an anti-His antibody. (c) Bactericidal activity of tag protein against *E. coli* (left) and *S. aureus* (right). Diluted bacteria samples were incubated with tag protein for 1 h or 2 h and then spread on LB agar plates. Viability was recorded as CFUs/ml after incubation for 18 h (n = 3).

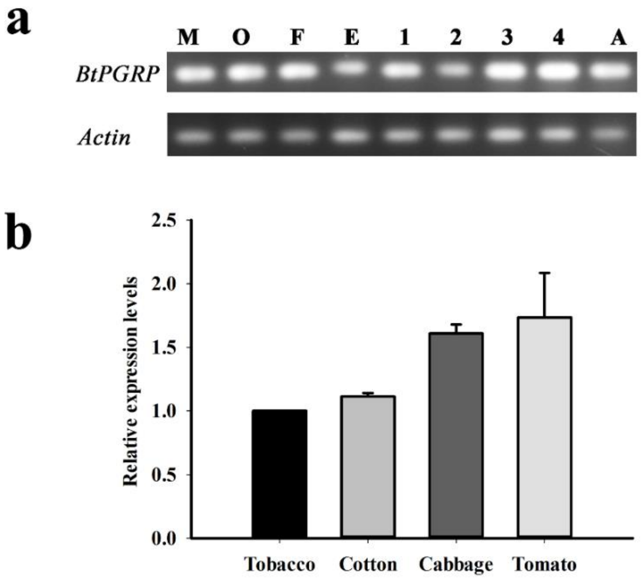

Figure S5. Analysis of *BtPGRP* mRNA expression. (a) *BtPGRP* mRNA expression in the the indicated whitefly tissues and life stages. M, midgut; O, ovary; F, fat body; E, egg; 1-4, 1st to 4th instar larvae; A, adults. (b) *BtPGRP* mRNA expression in the *B. tabaci* in response to host plants. Whiteflies feeding on different host plants (cotton, cabbage and tobacco) at least for three generations were used for RNA isolation. The data represent the mean ± SD of 3 biological samples.
